# A Guide to the Practical Study of Diseases of the Eye

**Published:** 1857-07

**Authors:** 


ART. II.—*A Guide to the Practical Study of Diseases of the Eye. With an Outline of their Medical and Operative Treatment.* By JAMES DIXON, Surgeon to the Royal Ophthalmic Hospital, Moorfields. Formerly Assistant Surgeon to St. Thomas's Hospital. London, 1855.

THE author of this manual thinks, in consideration of the fact that "so great an amount of talent and industry has of late years been devoted to the investigation of Diseases of the Eye, that a new volume on the subject should require a few words of introduction." It certainly can require nothing in the way of apology for its appearance, if it be one of merit. We cannot have too many works of *merit*, to guide the student of medicine in the management of the diseases of this most important organ; for it is an undeniable fact, that the profession at large is grossly ignorant on the subject: their stock of knowledge often at the best consisting in some crude ideas only of ophthalmia and cataract. With these they undertake to treat affections often requiring really consummate skill in their management; and yet, the profession is ever ready, and especially that part of it to whom, perhaps, the greatest sin is to be imputed, that in professorial robes, to raise an indignant voice against the credulity of the public in seeking aid of professing-advertising *oculists*, who reap a rich harvest from the neglect of the study of the eye affections by the profession at large. Much as we ourselves condemn quackery, we are forced to admit the fact, that these charlatans, although ignorant of general pathology and therapeutics, and often even of the anatomy and physiology of the organ, acquire, from the ample opportunities afforded them, a much larger amount of knowledge of the symptoms, diagnosis, and treatment of these diseases, and also, often, much more considerable skill in the operative procedures required for their relief, than is possessed by the majority of regular general practitioners. It would appear to have been long the custom with our profession to suffer the treatment of diseases of the eye to fall into such hands, for Jean de Vigo speaks of leaving the ordinary diseases of the eye to "*foreigners*;" which proves that our friend Jean was no Yankee, although he dubbed these interlopers with a favorite Native American epithet.

In the earliest ages of our profession, it would appear that the diseases of the eye received their full share of attention. Tradition tells us that there were full accounts of various diseases of the eye in the works of Thoth, who passed with the Egyptians as the inventor of all sciences and arts. These works were lost at some unknown period, but from tradition would appear to have been an enormous collection of more than twenty thousand volumes, written, probably, by different persons, and at various periods, and to have embraced all the learning of the ancient Egyptian priests, who were also the physicians. But we do not have to rely on tradition to prove that the Egyptians paid great attention to the subject, for Herodotus tells us of Cyrus writing to Amasis, king of Egypt, to send him an oculist.

The early Greeks were familiar with the diseases of the organ. To them we are indebted for the names which most of its affections now bear.

Chiron, the preceptor of Æsculapius, is reputed to have been skilful in the treatment of the eye; and Erisistratus, who lived in the courts of the

Syrian kings, concocted a great many eye-washes, to which the name of *panchrester* was given.

In the palmy days of the Roman Empire, ophthalmology and ophthalmic surgeons would appear to have flourished to a very great extent. More that fifty oculists are cited by Scribonius, Galen, and other old writers. And particularly in the times of Tiberius and Augustus Cæsar, it would seem that these specialists were as numerous, if not more so, than at the present time, and to have received great distinctions; for we find P. Attius Atimetus, styling himself *Augusti medicus ab oculos;* and Titus Syrius, claiming the same office to Tiberius. (Haller. Biblioth. Chir. vol. i, lib. i, § 24.)

It does not, indeed, seem unreasonable to suppose that the ancients acquired considerable proficiency and knowledge in the treatment of the eye. They were undoubtedly good and close observers; and although their ideas of the anatomy and physiology of the organ were very crude, and often erroneous, yet the importance and exposed position of this structure, the tangible character, as it were, of its affections, and the influence of the local applications upon them, not only furnish the best explanation for the great attention which the subject received, but afford strong grounds of inference, that the science made great advances in these early times.

We have positive proof of such progress having been made by the ancients, for besides frequent references to the diseases of this organ in the writings of Galen, Hippocrates, Pliny, Oribasius, and Ætius, we find in the works of Celsus, a full account of the state of the knowledge on the subject at his time, which shows clearly the great progress which it had made. Most of the diseases of the organ with which we are now familiar, are enumerated by him, and many of the operations described by him with great accuracy, would be thought very appropriate, in the opinion of any surgeon of the present day, for the cases for which he mentions them.

Mr. Dixon, as we have quoted above, alludes in his introductory remarks to the great amount of talent and industry which has of late years been devoted to the investigation of diseases of the eye. Few are aware of how very recent a date this devotion of a great amount of talent and industry to the subject is. Yet this fact is very readily proved by a simple reference to the dates of the publication of the various treatises on the subject which are esteemed of any value. A glance at the Bibliography of Diseases of the Eye, will show how very few treatises were published on the subject before the beginning of the present century, and of these few, how comparatively small is the number which can claim the merit of being anything more than books intended rather for public inspection, and notoriety, than for the advancement or diffusion of knowledge in the profession.

It is not easy to determine who wrote the first distinct treatise on Diseases of the Eye. Of the host of oculists whose names have been handed down to us through the earlier writers on medicine and surgery, we have no evidence, either traditionary or otherwise, that any one wrote a distinct treatise on this special subject. We should, indeed, be totally ignorant of the names of a large number of these gentlemen, were it not that they have been prefixed to formulæ for collyria, with many of which we doubt not those to whom their invention was thus imputed, were entirely unacquainted. Thus we read in Celsus of the Collyria of Philletes, Philo, Cleon, and many others, whose fame as oculists would never otherwise have reached us.

It is not before the seventh century that we can discover any evidence of the existence of a special work on the eye. This was said to have been written by Alexander Trallianus, who lived to an advanced age, and died in Rome, after having composed a number of treatises on medical and surgical subjects, one of which was on the eye. M. Eloy, author of the Dict. Hist. de la Médecine, Anc. et Mod., states, that the one on the eye, if it ever existed is not now extant. But Mr. Haynes Walton not only mentions this work of Alexander of Tralles, but states that it consisted chiefly of formulæ for collyria. It is frequently cited by Ætius and later writers.

There would appear to have been a number of treatises written on the subject by the Greeks and Arabs who immediately preceded Rhazes, few, if any, of which are now extant. Rhazes quotes from them. He refers to Lororacos de Morbis Oculorum, and to Jasue, Bin Thalnis, Sœpius, Serapion, and others, as having contributed to the literature of the subject. Rhazes himself may be said to have written a distinct treatise on the eye; for the second book in his Contenens is devoted to the diseases of that organ. The same may be said of Paulus Ægineta, who preceded Rhazes, and flourished about a hundred years after Alexander Trallianus, for his third book is exclusively taken up with the consideration of "some of the diseases of the eyes."

We have to pass from the times of Rhazes, in the tenth century, over a period of full a hundred years, before we can find even any record of the production of another treatise on the eye. It does not deserve the name of a new work. It was the production of Constantinus, surnamed Africanus, a native of Carthage, who, having travelled in the East in the search of knowledge, returned to his native place to pursue his profession there, but was driven out from it under fear of death, as he was looked upon as a magician. He took refuge in Apulia, and afterwards became a professor at the famous school of Salerno. From this post he retired into a monastery, and there indulged his taste for study, and composed, amongst others, a treatise on the eye, which however was, as we have intimated, but a compilation from the Greek and Arabian writings. Indeed, most of the medical works of that period were but compilations of the same sort. The blind deference to the ancients, and consequent want of confidence in personal observation, were the great causes of the want of progress in this age of ignorance. In none of the works, from the 6th century down, we might safely say to the 18th, certainly to the 17th century, can we find any evidence of progress as regards diseases of the eye. The works published in this period consisted chiefly of repetitions of what Celsus and others had written, interspersed with recent and often very crude errors. After the times of Constantinus, medicine and surgery rather receded than progressed, and we have to wander over another hundred years before finding another treatise written on the diseases of the eye.

Canamulasus de Baldach, a Saracen, who lived in the early part of the 13th century, published a book, on the articles from which remedies for the eye were to be prepared, and this is the only new treatise which appeared between that of Constantinus in the 11th, and that of Benevenutus Grapheus, at the close of the 15th century. About the same time with the appearance of Grapheus's work, that of Jesus Hali, on the Knowledge of the Infirmities of the Eye and their Cure, made its appearance. These treatises were published, together with the Chirurgiæ of Guy de Chauliac, in 1499.

The knowledge of diseases of the eye began to make progress, as did that of all other branches, soon after the great impetus given to the study of anatomy by Vesalius, Fallopius, and Eustachius, in the 16th century, and we consequently find the number and character of the treatises on the subject changed. We find appearing, in the beginning of the 16th century, the works of Mercurialis and Stephanus; and in 1602, edited by his son, Heurnius on Diseases of the Eye. This work belongs to the 16th century, for its author, the distinguished professor of Leyden, died in 1598, at the age of 55 years, of stone in the bladder. Soon after the publication of this work of Heurnius, there appeared Plempius's Ophthalmographia, and the works of Otto and Meibomius on the eye. It was in this, the 17th century, that the true nature and seat of cataract were first surmised by Rolfink, and demonstrated by Brisseau; and it was also in this century that we find enrolled such names as Kepler, Scheiner, Des Cartes, Ruysch, Mariotte, and Leuwenhoek, as contributing to the advancement of the knowledge of the eye; and yet, neither in this century, nor even in the first half of the 18th century, do we find (with a few exceptions) the treatises on the diseases of the eye assuming a truly scientific character. For, although before the close of the 18th century, the pathology and treatment of the eye had made considerable advance, and had received no little attention from men like Cheselden, Petit, Heister, and Sharp, yet the treatises on the diseases of the eye, with the exceptions of those of Maitre Jean, St. Yves, and Janin, consisted merely of formulæ for eyewashes and accounts of wonderful successes in treatment, published for the purpose of attracting custom.

The progress made in the 18th century is by no means to be judged of by the character of the *treatises* on the subject, or even by anything contained in them. The contributions made by those whose names we have referred to, and by many others, who devoted to diseases of the eye the attention they deserved, are not to be found in treatises, but in monographs and communications to learned societies. It is to the latter, however, that we must always look for the signs of advancement. An author of a treatise is too often biassed in his judgment, or possessed of peculiar views of his own, which prevent his giving a just *résumé* of the subject.

The present century has, it may be fairly said, been more prolific in treatises on the eye, than on any other speciality. Their name is legion, and those who are directing their attention to the subject, may be said to be innumerable; men are no longer in many places deterred from doing so, by the fear of the sneering epithet of "specialist" being attached to their names. A class of diseases which could be deemed worthy of special attention by such men as Scarpa, Travers, and Lawrence, cannot be beneath the notice of humbler men. All the greatest surgeons the world has produced, have found interest in the subject, and prided themselves on their skill in the delicate operations on the eye. Many, it is true, in former days, were deterred by public prejudice from paying proper attention to the subject; but in England, at least since Mr. Travers' time, the fear of being disqualified in public opinion, by a reputation acquired in diseases of the eye, for the treatment of other diseases, no longer deters surgeons from the cultivation of such a large and legitimate field of observation. Such a *special* reputation has, on the contrary, often since then served but as a stepping-stone to more general surgical practice. And it is but right the profession in this country should be aroused to a proper feeling on the subject, for with its members as much as with the public rests the fault of fostering a prejudice, if any exists, against paying the proper attention to diseases of the eye they deserve; and it is hard to conceal the fact, that such prejudice does exist to very great extent in this country. We attribute, we say, much of this fault to the profession at large. For when called upon to treat diseases of the eye, most practitioners, too little informed on the subject, either tamper with the patient, or confess candidly that they know nothing on the subject; assigning in either case as a cause for such a state of things, that the disorders of the eye are so very numerous and intricate, and the operations for their relief require such exquisite skill, as to need one's exclusive attention, to the utter disregard of all other diseases of the body; and hence the reasons of their neglecting them. Popular prejudice is thus fostered by those who should be the very ones to do away with such prejudices, for such prejudices are highly injurious to the science. It is true that affections of the eye must be studied to be understood, but they are not so peculiar as to differ in their nature from the morbid actions in other parts of the body. On the contrary, they are governed by the same laws, and influenced by the same remote sympathies. Hence, one must be a thorough surgeon to treat them properly; but how few will be willing to acquire a reputation for skill in such diseases, if it will act injuriously to him in general practice. Can it not, therefore, be readily seen, how many men of talent and industry may be deterred by this popular feeling, fostered by the profession itself, from devoting a *proper share* of their time and attention to the disease of this important organ? And thus this branch of medical science is made to suffer by the medical profession itself. We would repudiate the name of *oculist*, but we would have every physician thoroughly skilled in the treatment of diseases of the eye. We would have every one pay that attention to the subject which its importance demands. Ophthalmic surgery should be made a part of the regular course of study of every medical man in this country, as it is made in some places abroad.

We believe that the state of the literature of a subject may be generally taken as a fair criterion of the absolute amount of attention which that subject is receiving, especially in medicine, where all progress in science has been faithfully chronicled, with very few exceptions. If we were to judge of the amount of attention which diseases of the eye are receiving in this country by this criterion, we have but little to boast of, for of the only two works by American authors, with any pretension to originality, Frick's and Littell's, the latter is the only one that has a right to such a claim, for the former is but a mere compilation from Beer's elaborate treatise. Although it is not our purpose to enter with any detail into the merits of any other work on the diseases of the eye than Mr. Dixon's, we may remark in regard to Dr. Littell's Manual, that it has been deemed worthy of republication in Great Britain, where we have had an opportunity of knowing that it is very highly prized, and that if it is the only original work on the subject of which the country can boast, it is one of real merit. As a writer in the British and Foreign Medical Review observed: "It is no small triumph to Dr. Littell to be able to say that he has introduced almost all that is valuable, and everything absolutely necessary to the student, within the compass of two hundred and fifty small pages."

Mr. Dixon's Guide bears a very close resemblance to Littell's Manual, and has, we believe, but one merit over it,—it is a more recent publication. It is more than ten years since the last edition of Dr. Littell's Manual made its appearance; we hope, ere long, to be able to chronicle the issue of another, revised by its able author, who has for nearly a quarter of a century been one of the kindest and most skilful surgeons to the oldest ophthalmic institution in the country.

Mr. Dixon speaks of diseases of the eye as he has seen them, and has avoided, as much as possible, all theoretical and controversial matters. In respect to treatment, he has limited himself to general rules, and briefly and plainly to record what his own experience has led him to prefer. Such, he states in his preface, has been his aim. How well he has succeeded, we shall endeavor to give our readers the means of judging for themselves. The objects he has had in view should commend his work to all. He has certainly set out with the only motives which an author should have, in adding another to the many treatises which have so recently appeared on the subject. The great fault we shall have to find with his book in the following review, is that he has written too little. The necessity for condensing has, in too many instances, compelled him to pass too hurriedly over points to which, we think, more importance should be attached in a Guide to the Study of Diseases of the Eye.

In the first chapter of his book, Mr. Dixon describes the various modes of examining the eye; he gives special directions for the examination of the cornea in children suffering with scrofulous ophthalmia, &c. His description of the manipulation to expose the conjunctiva of the upper lid, by everting the part, is very clear. In reference to this point he justly remarks, that it "requires a good deal of knack."

He then passes to the examination of the parts behind the pupil, and to the investigation of the deeply seated structures of the eyeball, and in connection with this subject, he refers to the recent invention of the ophthalmoscope, by Helmholtz, and its modification by Ruette and Coccius. He describes the simplest form of the instrument, that of Ruette, and gives directions as to the manner in which an examination of the eye with it is to be conducted. In reference to this last point, he makes the following remarks:

"One very important fact should never be lost sight of by those who employ the ophthalmoscope, namely, that the mere concentration of powerful light on the retina, if continued for more than a few seconds, does of itself place the part in an unnatural condition. In exploring the internal ear, by means of artificial light, we may, indeed, concentrate the rays upon the tympanic cavity or its membrane, to any amount, without injury to the parts illuminated; but the *retina*, so far from being a merely passive object of examination, is just the one tissue in the body which appreciates the intensity of the rays which fall upon it; and it must be borne in mind that the eye may be irritable and intolerant of light to an extreme degree, even although there may be a considerable diminution in its power of perceiving objects."

Although we are willing to admit that the retina is not "a merely passive object of examination" with this instrument, and that it is "just the one tissue of the body which appreciates the intensity of the rays which fall upon it," still we cannot agree with the author in forbidding the use of the instrument for more than a few seconds at a time. The consequences dreaded from this concentration of light on the retina, have deterred many from using the instrument altogether; and this dread has even induced some to condemn it in toto, without ever giving it a trial. They also urge that, even admitting the use of the instrument *for a few seconds* to be attended with no evil consequences, the time is too short to allow of any satisfactory results from such an examination. We confess that, from our first impressions of the character of the ophthalmoscope, we were induced to participate in these evil prognostications in regard to it; but after a frequent, free, but careful use of the instrument for a period of more than two years at a large eye infirmary, where we have employed it certainly in more than one hundred different cases, in that period, we are convinced that these objections are more theoretical than real. We have employed it in cases of hyperæmia of the retina, acute retinitis, choroiditis, and posterior ophthalmitis, and we have *never* seen any evil consequence follow its use; and in not more than half a dozen out of the whole number of cases in which we have used it, have we found the patients to complain of the intensity of the light, although the examinations were often continued long enough for us to sketch the diseased structures, or to enable a few professional friends to view them after us.

We do not wish to be understood as advocating an indiscriminate and reckless use of the ophthalmoscope; we are even free to admit that there are cases, where its use, when long continued, may be attended with harm; but these, we are satisfied, are relatively infrequent. We are convinced that the instrument has in some quarters met with unmerited condemnation. This we believe, in some instances, has been from a want of proper knowledge and skill in its use. An optician mentioned to us, some time since, a circumstance which goes to confirm this conviction. A medical man, he said, consulted him about the manner of using a form of the instrument, which he had received through a friend from abroad. As the optician was as little familiar with it as the doctor, the two together made little, indeed no progress in its use, and they came to the conclusion, a real Yankee one, that it was a *humbug*. Not long afterwards there appeared in a medical journal a review of a work on Ophthalmic Surgery, from the pen of this same medical gentleman, in which the ophthalmoscope was condemned, as both useless and injurious.

The ophthalmoscope has to share the fate of every other innovation in our art. Vaccination was decried from the pulpit. The stethoscope was spoken of as a stick of wood with a fool at one end and a knave at the other; and the use of anæsthesia has been condemned as a defiance of the will of the Almighty, who but justly inflicts pain on poor mortal man. Yet all these boons to our profession have outlived their detractors, and are now treasured by us as invaluable; so, we doubt not, will be the fate of the ophthalmoscope. Ophthalmoscopy is but now in its infancy. As improvements are made, as they no doubt will be, in the construction of the instrument, and as greater skill and accuracy are acquired in its use, we will find it to become invaluable in the study of deep-seated disease of the eye.

At present we will not claim for the instrument any more value than Mr. Dixon assigns to it. He says:

"The chief value of the ophthalmoscope seems to consist in enabling the surgeon to set aside, as positively hopeless, a large number of cases formerly termed amaurotic or 'nervous,' which were assumed to be still curable, because their real nature could not be demonstrated.

"We now know," he continues, "that total disintegration of the vitreous body, detachment of the retina from its connection with the choroid, and other equally hopeless conditions of structures essential to vision, may exist without any alterations being produced in the outward appearance of the eye. In enabling us, therefore, to appreciate these conditions, the ophthalmoscope has proved of immense value."

We cannot, however, further agree with the author, when he says, that "the more delicate changes occurring in the early stages of deep-seated disease, whilst they elude detection by this instrument, are almost certain to be aggravated by its application;" for we are not so satisfied as to the certainty of this aggravation, when the instrument is judiciously used. We do not advocate an indiscriminate resort to it on all occasions; for we fully agree with Mr. Dixon in his remarks upon "subjecting persons complaining of slight defects of vision, to the searching glare of the ophthalmoscope."

"First," he says, "let the cornea be most rigidly examined, to ascertain whether some loss of transparency may not exist in the axis of vision. Then, after the pupil has been dilated, let the lens undergo a similar scrutiny; and until the observer has thoroughly satisfied himself that both these structures are transparent, let him not subject the retina to the possibly irritating effects of concentrated light."

The instrument in improper hands can, we doubt, not, be made to do harm. It requires "knack" in its use. Many a poor patient, we are satisfied, has had his eyes irritated by the long-continued, but often unsuccessful efforts of the tyro, to bring the instrument into adjustment on his eye. It requires long practice to obtain freedom and facility in its use; hence the author very properly advises that the student's "first trial should be made on one of the lower animals—a kitten, for example; and when he has acquired readiness in using the instrument, he may next proceed to examine patients who have long been hopelessly blind, but in whom the media of the eye remain transparent." (p. 8.)

In a subsequent part of the work (Chaps. IX and X), Mr. D. speaks of the healthy appearance of the retina and deep-seated structures to be observed by the instrument, which he describes accurately and even somewhat minutely; and also of some of their various pathological changes, which give rise to those physiological and objective symptoms, which have been heretofore classed under the vague term of amauroses.

He says, in reference to these latter, that "the earlier stages of disease (which alone are *curable*), produce changes so slight and delicate as to elude observation."

This, we are convinced from personal experience, is not really the case. We have met with some cases, at least, of congestion of the optic nerve, retina, and choroid, which we could detect by the instrument, and which have proved *curable*. Many such cases have been reported by German and other writers. But we have dwelt already long enough on this subject, and must pass to other points in the book equally worthy of careful consideration.

Chapter II is devoted to the consideration of the diseases of the conjunctiva, including Pinguecula, Pterygium, Simple Ophthalmia, Pustular Ophthalmia, Catarrhal Ophthalmia, Purulent Ophthalmia, Granular Conjunctiva, Gonorrhœal Ophthalmia, Purulent Ophthalmia of Infants, Scrofulous Ophthalmia, Exanthematous Ophthalmia, and Chronic Ophthalmia. The discussion of all these various and important topics is embraced in a space of only forty-seven (47) pages. This, perhaps, is as much as could be properly allotted to them; but we ourselves wish Mr. Dixon had written more fully, especially on the treatment of these affections, as they are so little understood by the mass of the profession; and in reference to which, all that he has said seems to us so judicious.

We have enumerated above the various forms of conjunctivitis of which Mr. Dixon treats, and it may be observed that his classification is simple. It corresponds to that which is most generally adopted by English authors, and is much more satisfactory than the various subdivisions resorted to by the French and German systematic writers on Diseases of the Eye.

The distinctions which the latter draw are so numerous, that they cannot but fill the minds of students with dismay, at the great variety which they suppose affections of the conjunctiva may assume; whereas, most of their subdivisions of conjunctivitis are, as Mr. Dixon observes, "purely fanciful, and wholly inapplicable to practice."

In reference to the treatment of that most common and often troublesome affection of the eye, catarrhal ophthalmia, Mr. D. makes a very proper discrimination as to the use of nitrate of silver; we quote his remarks in full on the subject. He says:

"It is in this purely conjunctival form of catarrhal ophthalmia, that the local application of nitrate of silver is of such remarkable utility. Where the sclerotic is much injected, or there is any rheumatism present—as evinced by the pink zone around the cornea, tenderness of the globe, pain about the orbit, and neuralgia throughout the opthalmic branches of the fifth nerve,—the nitrate of silver is contraindicated, at least until after the affection of the fibrous tissues has been subdued by appropriate treatment." (p. 23.)

There is no doubt, in our mind, that it is from a want of a proper discrimination in these cases, that many practitioners not only often fail to afford relief in catarrh of the eye, but absolutely cause destruction of the organ.

The strength of the solution which, Mr. Dixon says, should be used in catarrhal conjunctivitis, is two grains to the ounce of water; and this he recommends to be applied to the conjunctiva twice or thrice a day. He does not speak of the use of any other applications or collyria, nor does he make any reference to the necessity for a resort to depletion, either general or local, as is advocated by Lawrence in plethoric young persons.

Mr. D. seems to rely almost entirely on the local treatment of this affection. In this respect he differs very widely from Mr. Lawrence, who seems to attach the first importance to the general treatment, and to consider local applications of minor consequence. In this matter, however, Mr. D. has the authority of Mackenzie and Beer to sustain him, and we believe his plan of treatment will meet with the approbation of every practical ophthalmic surgeon of the present day. Still, although he relies on local means, he would not have the practitioner overlook the patient's general condition, for he says:

"Due attention must, of course, be paid to the state of the patient's general health; the bowels being kept open, and the diet regulated according to circumstances."

In reference to the last point, we think his remarks very judicious, and would be instructive to those who hope always to cure this affection by starvation. Here is what he says:

"As a rule, a moderately nourishing diet, and at least the accustomed quantity of beer or wine, are to be insisted upon. But as in a feeble and depressed subject an additional quantity of stimulus will hasten the cure, just so must a reduced style of living be enforced upon the overfed and intemperate. The common sense of the surgeon must teach him to keep his patients on the proper level in these matters, and not to stuff or starve them indiscriminately, in accordance with any scientific theory of disease." (p. 24.)

In his treatment of the purulent form of the disease, Mr. Dixon resorts to the early and free use of tonics, in place of the depletory plan formerly so much in vogue, and which he considers (and very justly, we think) cannot be too strongly condemned. He quotes the following startling statement from Mr. Wardrop: "The only case" (of gonorrhœal ophthalmia) "he had seen in which the eye was saved, was that of a young woman, in whom venesection was repeated as often as blood could be got from the arm. She lost 170 ounces in a few days, and looked as if every drop of blood had been drained from her body; the skin having nearly the hue of a wax candle."

This Mr. Dixon quotes to show the extreme measures formerly resorted to in the treatment of this dangerous disease, and then aptly remarks: "Can we wonder that thousands of persons, with that tendency to rush into extremes, which is one of the infirmities of our nature, should seek refuge from such treatment under the milder discipline of homœopathy?" (p. 32.) He further says:

"If the treatment of purulent ophthalmia by excessive depletion be judged by its results—the only sure test—we shall, I think, be forced to confess that there was ample cause for trying some less violent means of cure. It has been suggested that the more temperate habits of the mass of the people at the present day, as compared with what existed fifty years ago, may exert a considerable influence over the inflammatory manifestations of certain diseases, and that those surgeons who described purulent ophthalmia, as they saw it at the commencement of the present century, had really sometimes to contend with a greater fulness and force of circulation in their patients than we are in the habit of witnessing, especially among the overworked and crowded population of our great towns. Certain it is, that as far as my own experience at a large metropolitan hospital enables me to form an opinion as to the general condition of patients suffering under purulent ophthalmia, I should say that they are uniformly more or less depressed, with a pulse more feeble than natural, and in a state which in every way contraindicates general bleeding, and calls for the administration of tonics. There is usually a coated tongue, with loss of appetite; and a brisk purgative is needed at the very outset of the treatment. Afterwards, either bark and ammonia or quinine, should be given, and hyoscyamus, if the patient be restless. Pure air, to many the best of all tonics, must, if possible, be obtained; and all unnecessary confinement to bed or to one room, avoided. Meat may be allowed once a day, and a moderate quantity of beer or wine; but on this head, no arbitrary rule can be laid down. The surgeon's judgment must guide him as to the cases in which he ought to forbid stimulants, recommend them in moderation, or even insist upon an extra quantity being taken." (pp. 32 and 33.)

Mr. Dixon says nothing in reference to *local depletion* in the treatment of purulent ophthalmia, and his silence on this subject in connection with his strictures on the depletory plan of treatment may readily be construed into condemnation of a resort to such means. We hope, however, that it was not his intention to be so understood; for, although we would heartily join him in his philippic against general bloodletting in the majority of cases of this disease, we should be sorry to be denied the liberty of resorting to leeching and cupping in the treatment of such cases, and especially to the former remedy. Local depletion is not inconsistent with a free use of tonics and stimuli, and we are satisfied that many who employ the general tonic course of treatment will sustain us in the conviction, that they have (as we have), seen great benefit attending local depletion in connection with such treatment. We are sure we have seen the cornea saved in many cases of purulent ophthalmia by an early and free application of leeches, in conjunction with the use of tonics, which could not have been preserved without a resort to them. The amount of blood removed by the application of a few leeches to the neighborhood of the diseased structures is not such as to materially influence the general circulation, but the local effect in the conjunctiva is immediately apparent. The cornea in all violent conjunctival inflammations suffers by *default*, as it were, it being dependent for its healthy nutrition on the freedom of the vascularity of the neighboring tissues, and when the circulation in them is impeded, it must suffer.

Our author very justly endeavors to impress upon the minds of his readers, that the danger to the cornea should be the source of their greatest anxiety in the treatment of this disease. His remarks are well worth quoting:

"The student," he says, "ought constantly to bear in mind that, although the disease termed purulent ophthalmia, has received its name from that symptom which most readily attracts notice—namely, the profuse conjunctival discharge, the real source of danger lies in the *cornea;* and that, even if it were possible so to drain the patient of blood as materially to lessen, or even wholly arrest the discharge, we might still fail to save the eye. It is not the flow of pus or mucus, however abundant, that should make us anxious, but the uncertainty as to whether the vitality of the cornea be sufficient to resist the changes which threaten its transparency. These changes are twofold,—*rapid ulceration* and *sloughing*. Now, has any sound surgeon, I would ask, ever recommended excessive general bleeding and salivation, as a means of averting these morbid changes from any other part of the body except the eye?"

Mr. Dixon's use of the word *general* to qualify his objection against bleeding, may admit of the supposition that his strictures on the subject were only in reference to that mode of depletion. We, for our own part, hope such to be the case.

After purulent ophthalmia our author directs attention to granular lids as a sequel of this affection, as also of long-continued catarrhal ophthalmia. Why he should have interposed this section between those of purulent ophthalmia and gonorrhœal ophthalmia, we cannot see. It cannot be that he does not consider granular lids as ever occurring as a sequela of gonorrhœal ophthalmia. Mr. Dixon is too much at home with his subject to induce us to suppose such was his intention, and yet the arrangement is one that might readily lead the beginner into such a belief.

In this section on granular lids (which consists of only two pages and a half), the true nature of these granulations, so called, is pointed out to be "merely the mucous follicles and papillæ of the membrane enlarged by inflammatory deposits." He speaks of the size and appearance of these "granulations," their influence on the lid, acting, as they do, like foreign bodies, and of the various plans in use for their removal. In reference to this latter point he observes:

"I believe that in most cases of granular lid, our chief dependence must be placed in improving the patient's general health, by giving him iron and quinine, singly or in combination, regulating his diet, and, if, possible, placing him in a pure and bracing air. An issue in the skin of the temple, kept open with a single pea, and occasionally stimulated, if the discharge becomes scanty, with some caustic or other irritant, is a slow but often very serviceable adjunct. Tincture of iodine, painted on the skin of the lids, is also useful."

On the subject of the local treatment the following contains a summary of our author's views:

"I have," he says, "at various times, tried all the most approved lotions and drops, but have never satisfied myself that any of them were of much benefit. The acetate of lead in fine powder, dusted over the everted lid, produces considerable pain at the time of its application, but afterwards gives decided relief, apparently by mechanically filling up the interstices of the granulations, and so producing a smooth surface for the eyeball to move upon. As the salt slowly dissolves, it probably exerts also an astringent effect upon the vessels supplying the enlarged follicles and papillæ, and so diminishes the size of these excrescences." (p. 36.)

Granular lids is undoubtedly one of the most frequent sequelæ of long-continued conjunctival irritations. They form the essential lesion of three-fourths of the cases which present themselves at the various Eye Infirmaries of our Eastern cities, and in the Far West, they have become so exceedingly numerous, as to attract the attention of all—of the law as well as of the profession,—who may visit those garden lands of our Confederacy. Our author says that, "Any person who has attended the practice of a London Eye Infirmary, must have been struck with the fact, that the severer cases of granular lids, with the attendant deformity of entropion and misplaced eyelashes, are met with among the most destitute Irish patients." With us, on this side of the Atlantic, although there are to be found many aggravated cases of granular lids from the Emerald Isle, still, as far as our observation goes, the worst cases we have ever seen, were from the other side of the mountains, and amongst those who had never even beheld the deep waters of the Atlantic. In the great Northwest, and in the Valley of the Mississippi, contagious ophthalmia appears, as far as we can learn, to be an endemic disease in certain districts; and these are the sources of the most aggravated cases which have fallen under our notice; and we are sure all Mr. Dixon's American readers will look especially to this part of the book, to see if he has anything to teach them on the subject.

The pathological lesion of "granular lids" was not known to the earlier writers on Ophthalmic Diseases. Its sequents of entropion, pannus, and chronic corneitis, in those who had suffered with Egyptian ophthalmia, had all been well observed, but their true cause had never been detected, from the fact that, before the time of Ware, who flourished in England about the beginning of the present century, no surgeon seems to have known how readily the lids could be everted. Ware was the first to describe this manipulation; but although he pointed out the mode of exposing the greatest seat of these granulatious, the honor is not due to him of having first detected them. This belongs to John Cunningham Saunders, the founder of the London Ophthalmic Infirmary.

The latter surgeon has described them as though they originated from an ulcerated surface—that they were indeed *granulations;* and he has the credit of having given them this very name. His plan of treating them was just such as was resorted to in the management of exuberant granulations, that of excising them.

Since his time, the disease has been generally recognized. But with many its true nature, and more especially its causes, have not been properly understood.

Thus, some writers speak of these "granulations," as though they were really *granulations;* and others, as though they arose from one peculiar form of ophthalmia, or that they were attributable to the use of certain local remedies.

Thus Nelaton speaks of them as pathognomonic of Egyptian ophthalmia.

Lawrence has said that the disease "almost deserves to be named from its exciting cause, lunar caustic ophthalmia;" and Mackenzie, although inclining somewhat to Lawrence's views, believes, "neglect of proper treatment and especially of depletion, general and local, in the early stage of the puro-mucous ophthalmia, is the most frequent cause of this affection."

Our author does not commit himself very clearly on these points, which we think important, for, if the true nature and causes of this troublesome affection were properly understood, it would occur less frequently than it does. He speaks of the disease as the result of "purulent ophthalmia, and long-continued ophthalmia of a severe character," but does not deem it important to explain how these diseases result in such a state. His old master, Frederick Tyrrell, is so clear on this point, that we cannot refrain from quoting his remarks on the subject. He says:

"I have shown that these affections, purulent and catarrhal ophthalmia, commence on the palpebral division of the conjunctiva, and from thence extend to the ocular portion. They disappear in the contrary order; leaving first the ocular part of the membrane, and that in which they appear last, and linger in the palpebral portion of the tunic, or that in which they first appear, and, in this division of the membrane, the morbid action may remain in so trifling a degree, as to escape the observation of the careless practitioner, who may be satisfied with the perfect restoration of vision, independently of slight occasional interruption from a collection of superabundant secretion." (Treatise on Diseases of the Eye, vol. i, p. 124.)

We would also take exception to the summary way in which Mr. Dixon dismisses the various remedies, beside the acetate of lead, which have been used for the cure of the disease in question. There are certainly some which, from the great frequency with which they are used, if for no other reason, deserve to be at least mentioned by him. We should be glad to have found his opinion more definitely expressed; for instance, on the use of the sulphate of copper and nitrate of silver, which he does not even mention, but which are so generally used, as to demand some expression of opinion even in an outline treatise on diseases of the eye. There are, undoubtedly, cases in which these remedies, especially the sulphate of copper, will prove injurious rather than beneficial, and when Mr. Dixon's favorite remedy will be of great service. It seems, however, important to us, that these differences should have been pointed out to the student by Mr. Dixon.

Most modern writers on diseases of the eye, speak of three varieties of *granulations*,—those which resemble the surface of a healthy or healing ulcer, those which consist of soft, flaccid, somewhat pediculated villi of a pale-red color, and those which are hard, pointed, dull-red, and projecting but little above the surface; and these differences we deem important, as demanding a resort to different means for the cure of the disease. The first variety, we are satisfied, yields more readily to the lead than other means, and is aggravated by the sulphate of copper; the second, we believe, will be found to be more readily cured by the alternate use of a weak solution of the nitrate of silver, and the stick of copper; whilst the third nearly always, in our opinion, requires a resort to the solid stick of sulphate of copper.

Our author does not labor as some writers do, to prove that there are any signs by which we can distinguish gonorrhœal from any other form of purulent ophthalmia, or even catarrhal ophthalmia, at its onset; although he states that this form of disease is often, if not generally, more rapid in its development and progress than any other. He also, and justly, we think, takes exceptions to the doctrines of many writers, that gonorrhœal ophthalmia usually attacks one eye only, and declares that he does not remember to have seen a well-marked case of gonorrhœal ophthalmia in which both eyes were not affected. As to the frequency of the disease in either eye, or in either sex, our author observes his usual silence; and in reference to the sources of the contagion, or origin of the disease, he is almost as much reserved, for he speaks only of a source of the disease in the *vulgar* practice of persons bathing their sore eyes in urine. The balance of his section on this subject is taken up with the details of the history of a case acquired in this way;—where a young man, by the advice of *his mother-in-law*, attempted to cure himself of an attack of ophthalmia, from the introduction of dust in his eyes, by washing them with his urine, he suffering at the time with gonorrhœa.

Our author next discusses the subject of the ophthalmia neonatorum. Here he, at the very outset, endeavors to enjoin on the student the fact (which is so often overlooked), that the most important point, and one to which he should first direct his attention, is the condition of the *cornea*, its clearness or opacity, and not the more obvious appearances of redness, swelling, and purulent discharge, which the eyelids present. This is often not easy to accomplish; indeed, with the inexperienced it is almost impossible. Their efforts to separate the lids nearly always resulting in their simply everting them, and the tumid mucous membrane bulging through the commissure entirely obscures their view of this important part.

He also points out the fact, that some of the discharge having become coagulated by the use of astringent washes, may be found lying over the cornea, and resembles very closely a slough of that structure, and may, by a hasty inspection, be mistaken for such.

Mr. Dixon's plan of treating this disease consists, in the mild forms, in the frequent use of mild astringent lotions, applied by means of a syringe. But in the severe forms, he says all local means may prove unavailing, unless the child be well nourished; he therefore observes that the health of the mother, "and her ability effectually to suckle the child, are most important points for the surgeon's consideration."

He further says, that—

"Weakly infants are sometimes much benefited by giving them, daily, a few drops of Battley's liquor cinchoniæ in a teaspoonful of milk. Blisters," he says, and we doubt if he could say a truer thing, "only exhaust and irritate such subjects, and bleeding by leeches, even to the smallest extent, may destroy the last chance of arresting the progress of a corneal ulcer."

And finally, he observes, "to attempt bringing up by hand" a feeble infant affected with such ulceration, is to doom the eye to almost certain destruction.

*Scrofulous ophthalmia*, the form of disease of the eye characterized by the development of phlyctænulæ or pustules, Mr. Dixon considers a misnomer, for he says—

"Although it is very frequently met with in patients who afford decided evidence of a scrofulous constitution, it undoubtedly affects others who have never shown any such tendency; and again, the *cornea* is the tissue in which the disease especially manifests itself, not the conjunctiva, which the conventional meaning of the word '*ophthalmia*' would imply to be primarily affected."

Chapter III is devoted to a short notice of the subjects of ecchymosis, œdema, fatty deposit beneath conjunctiva, cysticercus telæ cellulosæ, and chemical injuries. There is only one subject in the last section to which we would especially call attention. In this section on chemical injuries, he speaks of the disfigurement caused by the injudicious use of nitrate of silver, consisting in an indelible brown or olive colored stain of the conjunctiva. "This disfigurement," he says, "will not take place under judicious treatment; for nitrate of silver, which is so invaluable in catarrhal and purulent ophthalmia, exerts its beneficial influence within a few days, and its use can never be required for more than a few weeks at the farthest." There is undoubtedly a great deal of mischief done in this way, by the incessant and injudicious use of the nitrate of silver, but we do not think that many who see much of chronic ophthalmia will be willing to admit that nitrate of silver, "can never be required for more than a few weeks at farthest."

Mr. Dixon says nothing of the use of iodide of potassium, or any other remedies applied locally for the removal of this unsightly discoloration.

Under the head of Diseases of the Cornea, our author first alludes to the subject of *conical cornea*. In reference to the pathology and real nature of this rare affection, he candidly confesses the obscurity in which the subject rests. He gives the *quietus* to Sichel's doctrine, that the deformity is *always* preceded by a *central ulcer*, by stating the fact, which he has repeatedly verified, that in some of the most marked cases not the slightest opacity could be found, either at the apex of the cone or elsewhere. He states, however, as the only point in the pathology of the disease of which there seems to be any certainty, that in every instance where the cornea in that disease has been examined after death, the central portion or apex of the cone was found thinner than natural.

In the section on inflammation of the cornea, the reader will find Mr. Dixon expressing his conviction, and that too, in a very decided manner, against the treatment of this troublesome affection by active depletion in the form of a general and local bleeding, and the administration of mercury so as to affect the mouth, which is so generally recommended by those whose opinions are entitled to the utmost attention, from their large experience and high scientific attainments.

Mr. Dixon's work will prove invaluable to the young practitioner, if it does no more than to restrain him in his disposition to resort to violent or active depletory means for the relief of this affection. The poor victims of this disease, badly fed, puny, fretting children, whose stomachs and appetites have been spoiled by indulgence, by the parents, in cakes and candy, to stop their crying; and who have been cooped up in all probability in a hot smoky kitchen, are not such, in our opinion, as are likely to be benefited by *depletion* and *mercurialization.* "Under the steady use of tonics, especially iron, with or without quinine, the inflammatory symptoms," our author says, in these cases, subside; the vessels which had begun to shoot into the cornea dwindle and disappear, the haziness is lessened, the irritability of the eye subsides, and it is gradually restored to usefulness. Counter irritation by means of repeated small blisters to the temples, or behind the ears is an important aid to the tonic treatment. "In certain subjects," he further observes, "a few leeches occasionally applied to the temples afford great relief, but in the majority of cases they are useless, or even injurious, and aggravate, instead of lessening, that neuralgic character which the pain so often assumes when the fibrous tissues of the eyeball are inflamed."

Following this section on inflammation of the cornea, we have one on inflammation of the cornea with suppuration; then one on ulceration and opacities of the cornea. This, we doubt not, will strike most of Mr. Dixon's readers as an unnecessary *splitting of hairs,* in a work where so much condensation is required to discuss fairly all the subjects embraced in it; but the substance of these sections is of the utmost importance to be well understood; and although much space might have been saved by considering them together, as for instance in the repetition of the author's remarks against depletion, and in favor of the use of tonics in the latter sections; still all that he says we deem so judicious as to bear slight repetition, and to be worthy of careful perusal by all his readers. To some prominent points in the last of these sections, we will now call the attention of our readers.

Passing over his remarks on the care often required in the detection of ulceration of cornea, we make the following extract:

"The edge of an ulcer in which the destructive process has ceased, and reparation begun, is always more or less hazy, and at the same time there will be found some vascularity of the sclerotic immediately adjoining the part of the cornea, where the ulcer is situated, and occasionally a fine vessel or a delicate plexus of vessels, may be traced from the vascular portion of the sclerotic to the border of the ulcer itself." (pp. 83–84.)

These remarks we deem of great importance, for, as our author observes, this vascularity is often regarded as an evidence of unhealthy action, whereas it is only the channel "through which the reparative material necessary to the filling up of the ulcer is conveyed to it."

When the ulcer perforates the thickness of the cornea, and *prolapsus iridis* ensues, our author says: "In some books on ophthalmic surgery, the reader is directed carefully to push back again, with a probe, such protruded portions of the iris; but he is not told how they are to be *kept* back." Nor does Mr. Dixon tell us how such an object may be attained. He seems, on the contrary, to consider efforts for such a purpose fruitless, and directs the surgeon to content himself with endeavoring "to produce speedy adhesion between the protruded iris and the edge of the corneal ulcer with which it lies in contact," "by a suitable tonic and stimulant treatment," and especially "by very carefully and lightly touching the parts with a finely-pointed stick of nitrate of silver."

We find nothing here in reference to the use of belladonna in such cases. The value of this remedy, so strongly insisted upon by Mackenzie, Tyrrell, and Wilde, deserved at least an allusion from Mr. Dixon. We have seen it of great service where the perforation of the cornea was but slight and recent. In such instances, its influence on the iris has been such as to cause the protruding portion to recede.

The frequency of corneal opacities is so great, and their deformity such, as to attract the reader's attention to the portion of Mr. Dixon's work devoted to their consideration. His views on this subject, although containing nothing new, are judicious, and consistent, as the rest of the work is, with the principles of a sound pathology. He alludes to the various forms and causes of these blemishes, and to the variety of stimulating drops, ointments, and powders which have been vaunted for their cure. In infancy, when the reparative processes are rapid and active, more or less transparency may be regained in some cases of opacity, and that, too, from ulceration. In such cases in adults, no such hope, however, can be held out to a patient.

"*Time*," Mr. Dixon says, p. 88, "I believe, is the great clearer of corneal opacities; and the surgeon who is consulted respecting those which follow recent ulcers in children, may comfort the friends with the assurance that as the children grow up, the specks—they can never wholly disappear—will become smaller and less noticeable, provided no fresh inflammation should set in. If, however, the speck is of old date, and its margin abruptly defined, it is useless to attempt its removal."

As to the probable result of treatment of opacities not dependent on ulceration, but merely the result of effusion in the substance of the cornea, our author is totally silent. Such opacities, so frequently the sequelæ of scrofulous corneitis or granular lids, are spoken of by most authorities as amenable to treatment in a very great degree.

The resort to *operative* interference for the relief of opacities of the cornea, does not meet with much encouragement from our author. Dieffenbach's operation of excising a central leucoma, involving the whole thickness of the cornea, and bringing the edges of the wound together, and Dr. Nussbaum's more recent but less rational procedure of cutting a hole through the cornea and inserting therein a small piece of glass, meet from him the condemnation they deserve. In cases of opacity of a superficial character, from the deposit of lead, Mr. Dixon does, however, resort to an operation of scraping off the opacity.

In connection with this subject, Mr. Dixon mentions an exceedingly interesting case of calcareous deposit between the epithelium and anterior surface of the cornea, similar to a case which was met with by Mr. Bowman, about the same date of Mr. Dixon's observation. The deposit (in both eyes) gave the appearance, on a cursory observation, of the absence of pupillary orifices in either eye. It was in the form of a transverse opaque band, passing along the equator of each cornea so as entirely to hide the middle third of the iris; they had a brownish tint, closely resembling the irides. They were very superficial, and when removed by Mr. Dixon and examined by Mr. Thomas Taylor, were found to consist of flakes of phosphate and carbonate of lime. Mr. Bowman's case did not present the brownish tint in the deposit, but that of ordinary nebula.

The concluding portion of the chapter on the cornea is devoted to injuries of that structure and their mode of treatment, and is a condensed summary of what is usually done in the management of such accidents. The only matter of novelty to be noted here, is Mr. Dixon's mode of procedure in cases where the fragment of iron or other foreign body transfixes the cornea and projects into the anterior chamber, but is too deeply buried to allow of its seizure with the forceps. The great risk of the fragment being wholly pushed into the anterior chamber during the attempts at extraction, makes the management of such a case exceedingly difficult, unless some such expedient as Mr. Dixon's is resorted to. He says:

"In such cases I have several times avoided this accident by first thrusting through the outer margin of the cornea, a broad cutting needle, and carrying it onward until its anterior flat surface could be brought into contact with, and pressed against the point of the foreign body." (p. 103.)

The sclerotic, its appearance in health, acute and chronic inflammation, staphyloma scleroticæ, and wounds and injuries of the sclerotic, form the subject of Chapter V.

The little brownish blotches near the cornea, from which small vessels emerge, are not regarded by Mr. Dixon as evidences of a congested state of the choroid, as they are thought to be by Dalrymple, Sechel, Mackenzie, and others, for he says he has repeatedly met with them in persons whose sight was perfect.

His enumeration of the symptoms of either the acute or chronic form of sclerotitis is very imperfect. There is no allusion to the constitutional disturbance so marked generally in the acute affection. Nor are any diagnostic marks given by which the disease can be distinguished from either inflammation of the cornea or iris; and these, we think, the author ought to have taken the pains to have furnished—having alluded to the fact that inflammations of these three structures are frequently associated together. The fact of attacks of sclerotic inflammation being so much modified by rheumatic complications, is the reason assigned by the author for not entering fully on the treatment to be pursued in the management of this disease. His chief dependence appears to be on quinine and purgatives, or colchicum. Blisters, in the acute form, aggravate, in his opinion, the neuralgic pains, which he believes are most effectually relieved by a liniment of chloroform and oil. Leeches, he says, only afford slight and temporary relief. Of general depletion in the acute stage he makes no mention whatever.

His remarks on the treatment of the chronic form are embraced in these few words: "In this chronic affection quinine is very useful; and blisters applied to the temple or behind the ear act most beneficially."

The treatment of injuries of the sclerotic, enjoined by Mr. Dixon, is very judicious; consisting "in maintaining the wounded part *in perfect repose*, both in respect to motion and light. A week or ten days is not too long a time for keeping the lids uninterruptedly closed without examining the injured part." In reference to ruptured globe, he says the surgeon "must not trust too much to energetic treatment;" for those cases seem to have done best eventually, where there was the least amount of interference with the reparative efforts of nature, but where the one essential, perfect repose of the injured organ, was secured. (p. 114.) The avoidance of mercury, a resort to anodynes, and the regulation of the patient's diet by the vigor of his circulation, are the general means indicated by nature and advocated by our author in the management of these injuries.

Chapter VI is devoted to the iris, its congenital defects and prominent diseases, and embraces very nearly fifty pages of Mr. Dixon's book. Of this chapter a large portion is, of course, devoted to the consideration of iritis. The varieties enumerated by our author are the traumatic, rheumatic, and syphilitic. Of the gonorrhœal and arthritic forms, mentioned by most authors, and especially by the German, Mr. D. is not aware of having even seen a single case; and he says, he would even hesitate to speak separately of scrofulous iritis, were it not for one patient, who came under his notice several years ago. It was in an ill-nourished boy, five years and a half old. The cornea was slightly hazy; the iris of a slate color, with dull red vessels running over its surface and above the pupil, and to its inner side lay an irregular, nodulated, yellow mass, precisely similar in appearance to the fibrin deposited in syphilitic iritis. Some yellowish fluid also lay at the bottom of the anterior chamber. There was no appreciable change in the form of the pupil.

"The treatment pursued in this case was rather dietetic than medical, in the vulgar sense of the word. Two or three grains of hydc. cret. were given at bed-time; and some liq. cinch. once a day, but good beef-tea, and other nourishing food, were chiefly relied on."

By this means a cure of the disease was effected within two weeks' time.

This case of scrofulous iritis, we consider exceedingly interesting, and we think it will prove so to all Mr. Dixon's readers on this side of the Atlantic, who, we are certain, have frequent opportunities of observing the disease amongst our colored population, either north or south of Mason and Dixon's line. We can produce from our own records, four or five cases observed in as many months lately. But the case will prove valuable to us, not only as directing our attention very forcibly to the peculiarities of the disease, but, from the very happy result attending Mr. Dixon's mode of treating such cases, and which we ourselves are satisfied is the only judicious one to be pursued.

But to return to our author's chapter on iritis. He draws attention to the very great difference in the amount of irritation attending wounds of the iris without disturbing the lens, and those that are accompanied by displacement of that structure. As, for instance, the simple incised wound, in the operation for artificial pupil, is followed by very little inflammation, whereas, in the operation for cataract by displacement, should the lens be left pressing upon the iris, it will give rise to an iritic irritation, which will prove almost uncontrollable. So, also, in accidental wounds of the eye, should the capsule and even the substance of the lens be penetrated by an object wounding the cornea and iris, and their position be undisturbed as regards the iris, but little if any more inflammation will occur than will be required for the repair of the injury; whereas, let the lens be displaced and encroach upon the iris, and it will keep up irritation in that structure as long as it remains there. It is true, it will be eventually absorbed through the aqueous humor, but until thus removed, it will act injuriously, and sufficient time may elapse before it is disposed of to do irreparable mischief. It was in consideration of the evil consequences following this displacement of the lens, that Mr. Barton, of Manchester, Mr. Dixon tells us, was induced to suggest and try the operation of extracting the lens, and he has related the good results of such a procedure in several cases. This operation, as Mr. Dixon says, to be of any service at all, must be performed early, otherwise, fibrinous adhesions form between the iris and the capsule of the lens, which would hinder the extraction of the latter. (p. 130.)

Mr. Dixon makes a remark in reference to the use of mercury in the treatment of traumatic iritis, which, we doubt not, will surprise some of his readers, but which we believe will be sustained by careful observation. It is, that it has little effect comparatively in the cure of such cases.

Our author's distinction between the rheumatic and syphilitic forms of iritis, is very satisfactory. As regards the treatment of the first, he advises when the sclerotic and cornea alone suffer, active purging, bleeding from the temples by leeches, and abstinence from stimulating drinks of every kind, then quinine or bark, with hyoscyamus. Mercury is the remedy to be given whenever there is any tendency manifested to fibrinous effusion in the iris, whether the inflammation be of syphilitic origin or not. He says:

> "Subsequently, the success of this treatment (with mercury), depends on its being begun at an early stage of the disease. The fibrin then exists as a mere unorganized secretion, and even if very abundant, rapidly undergoing absorption, but when it has become consolidated, and traversed with bloodvessels, it yields much more slowly to medical treatment," &c. (p. 137.)

There is not a modern writer heretofore, who has not recommended, and in the strongest terms, the free use of belladonna or atropine, in the treatment of the various forms of iritis, not only for the soothing effect produced on the peculiar neuralgic pain of the disease, but chiefly, too, for the purpose of preventing one of its most injurious effects, closure of the pupil; we doubt not, therefore, that most of his readers will participate in our surprise on finding that Mr. Dixon declares them useless during the active stage of the disease, and injurious at a late period. Our readers must be satisfied from the insight we have given them thus far of Mr. Dixon's work, that he is a reflecting man, and a close observer; and will be willing, therefore, we are sure, to give great weight to all that he may say, especially if it be at variance with the generally received opinions, for *we* feel satisfied, that he would promulgate no new views merely for the sake of their novelty, but from conviction of their truthfulness. His opinions in this matter are, therefore, entitled to some consideration. Here they are:

"Invaluable, indispensable as atropine is in our examination of many morbid states of the eye, I do not regard it as of any service in iritis; for, as I stated at the commencement of the section, an inflamed iris loses its power of motion. Atropine, therefore, must be *useless* during the active stage of inflammation. At a later period, when the iris is beginning to recover its motory function, it may, I think, even *do harm*, and in the following way: The hinder surface of the iris, termed "uvea," is covered with a layer of pigment cells. When fibrin is poured out behind the iris (which, no doubt, happens in all cases of acute inflammation), these pigment cells become, for a time, firmly united to the capsule of the lens; and if, when the iris is regaining its motory function, a forced dilatation of the pupil be effected by the influence of atropine, some of the pigment may be detached from the posterior surface of the iris, and left adhering to the capsule, forming those brown patches so familiar to us in patients who have suffered from iritis. Only get rid of the fibrin which is gluing the pigment cells to the capsule of the lens, and the iris is at once effectually liberated.

"People sometimes talk and write as if occlusion of the pupil in iritis were the result of spasm of some sphincter muscle, the contractions of which could be paralyzed by atropine, and the pupil thus kept permanently dilated. But the real cause of closure is totally different from this. Fibrin is poured out from the surface of the iris and edge of the pupil upon the front part of the capsule of the lens, overspreading the latter, where it corresponds to the area of the pupil. Now, if this effusion is not quickly removed by absorption, it becomes organized, and forms a membrane stretching across and blocking up the pupillary opening. Gradually this membrane contracts, and in doing so draws together the edges of the pupil, until that aperture is reduced, in some cases, to the size of a pin-hole."

Logical as all this may appear, are either the premises or inferences strictly true? Does the iris at the commencement of an attack of inflammation lose its power of motion entirely? Is the surface of the capsule of the lens, corresponding to the area of the pupil (unless that orifice is very small), overspread with lymph? and has that lymph a contractile power greater than that deposited on the posterior surface of the iris near its ciliary margin, and which can and does bind it to the edge of the lens? On the truthfulness of these propositions it seems to us that the correctness of Mr. Dixon's arguments hinges.

Had Mr. Dixon entered with any minuteness of detail into the symptoms characterizing iritis at the very outset of the disease (which he has not), we doubt not but that we could have convicted him of an error out of his own mouth, as regards the contractility of the iris in the acute stage of the disease. But the only expression he uses (and the one to which he refers in the quotation above), is "an inflamed iris loses its contractile power." This no one will deny, perhaps; but does no time elapse from the first manifestation of the disease till the intensity of the inflammation is such as to cause this loss of contractile power? Has Mr. Dixon never seen iritis in its incipiency, when the characteristic symptoms were sclerotic injection; tenderness of the ball; circumorbital pain; lachrymation; slight change in the color of the iris, and its motion *impeded*, "so that the pupil dilates and contracts slowly on the admission or withdrawal of light (Tyrrell);" and where, on comparing the two eyes, the one affected is observed to have its pupillary orifice smaller than that of the opposite eye, although it still has the power of dilating? Cannot belladonna or atropine be of *any* service in this stage of acute iritis? We certainly think it can, and we believe the experience of every surgeon will sustain us in such a belief. We therefore think Mr. Dixon has been too sweeping in his condemnation of these remedies. We agree with him that when the iris has become adherent to the capsule of the lens these remedies *will* prove injurious.

We must pass over what Mr. Dixon has to say in reference to the treatment of iritis, in which we find nothing worthy of special notice, although all he says we think judicious and well worth reading, and direct attention for a moment to Chapter VII, on Inflammation of the Iris and Cornea together. This disease constitutes what has been described by previous writers as Aquo Capsulitis, a name which, as Mr. Dixon justly says, ought to be erased from our vocabulary, as being based on false anatomy; there is no such structure as the capsule of the aqueous humor, a serous membrane and shut sac as it was supposed to be, and hence there can be no such disease as Aquo Capsulitis. Our author indicates by the name he has adopted the real nature of the disease, the symptoms of which he points out with conciseness and perspicuity. He states that its most frequent subjects are young persons, and that they require small doses of hyd. cum. cret. with tonics and iodine.

If the reader desires still further evidence that Mr. Dixon has only attempted to teach what he knows from personal observation, he need but to turn to his chapter (the eighth) on the choroid and retina, in which he will be struck by the author's extreme candor, in stating his inability to point out signs characteristic of inflammation confined to either of these structures. Now, inflammation of these structures has been described with so much minuteness, and their diagnostic signs detailed with so much precision by recent writers, especially the German and French, that this statement from one of Mr. Dixon's opportunities for observation, contrasts very strangely with theirs. Yet we believe it to be true in every sense. And we want no better evidence of Mr. Dixon's honesty of purpose than this confession.

We have ourselves frequently met with cases where there were none of the symptoms usually stated as indicative of disease of the choroid and retina, but which revealed by the ophthalmoscope most extensive changes in both these structures. "Such examinations prove," as Mr. Dixon observes, "how little dependence can be placed on those descriptions of 'choroiditis,' and 'retinitis,' as set forth by some systematic writers, who would teach us, from the condition of the pupil, the color of the iris, or the appearances noticed by the patient, to pronounce with certainty as to whether the choroid or the retina be the seat of disease."

We have before alluded to the contents of Chapters IX and X, the merits of which we should like to discuss more minutely if our space would admit of our doing so. But we have already exceeded the space assigned us for this review, and will not only have to pass over these, but also content ourselves with the simple enumeration of the subsequent chapters of the book, which we should have taken as great pleasure in examining as minutely as we have all the preceding ones.

Chapter XI, is on the lens and its capsule, and contains an account of the catoptric test; altered position of the lens; and the diseases of the lens, which, in Mr. Dixon's opinion, consists only of cataract; for this is the only disease of the lens he describes, or even alludes to.

Chapter XII is devoted to diseases which involve all the tissues of the eyeball, including, under this head, that very vague disease, glaucoma, scrofula, encephaloid, and melanotic deposit.

Chapter XIII contains some account of diseases of uncertain seat, namely, muscæ volitantes, long-sight, short-sight, and inability to distinguish certain colors.

Chapter XIV is on the lachrymal passages, their mode of examination, inflammation of the lachrymal sac, chronic distension, and treatment.

Chapter XV refers to the eyelids, entropion, ectropion, inflammation of the lids, hordeolum, tinea ciliaris, lippitudo, phtheiriasis, tumors, cysts, warts, and nevi, injuries, carcinoma, and epithelial cancer.

Chapter XVI, the orbit, diseases of parts surrounding and acting upon the eyeball, namely, protrusion of eyeball, and various displacements, orbital abscess, paralysis of third nerve, causing ptosis, &c., strabismus, affections of the oblique muscles, causes and treatment; and deformity following sometimes operations for strabismus.

The remainder of the work, including a little more than eighty pages, refers to operations, for cataract, displacement, depression and reclination, solution or absorption, operations on opaque capsule. The operation of extraction, for artificial pupil, for staphyloma, strabismus, and removal of eyeball; and these remarks, on the various operations in which, as the author tells us, he has confined himself to such a general description as may render the principles upon which they are founded intelligible to the student, are closed by some very judicious observations on the use of chloroform in operations on the eye.

Finally, the work contains a short appendix of cases, with some excellent woodcuts, and a plate of the instruments preferred by Mr. Dixon.

It can thus be seen by the reader, that Mr. Dixon has written an excellent manual on the eye; and we are sure that all who read it will regret that its author had not written more It is true, that by doing so, he would have increased the size of his book, and thus, perhaps, altered its character as a manual; but there are, nevertheless, many points, to some of which we have alluded, on which he might, and, we think, ought to have spent more time. We ourselves part with the book as we would with an agreeable friend, whose company we regret we could not enjoy longer.

